# Analysis of Postoperative Remodeling Characteristics after Modular Inner Branched Stent-Graft Treatment of Aortic Arch Pathologies Using Computational Fluid Dynamics

**DOI:** 10.3390/bioengineering10020164

**Published:** 2023-01-27

**Authors:** Fen Li, Yating Zhu, Hui Song, Hongpeng Zhang, Lingfeng Chen, Wei Guo

**Affiliations:** 1 College of Mechanical and Vehicle Engineering, Taiyuan University of Technology, Taiyuan 030024, China; 2 Institute of Applied Mechanics, Taiyuan University of Technology, Taiyuan 030024, China; 3 First Medical Center of Chinese PLA General Hospital, Department of Vascular Surgery, Beijing 100853, China; 4 College of Biomedical Engineering, Taiyuan University of Technology, Taiyuan 030024, China

**Keywords:** modular inner branched stent-graft, aortic arch pathology, computational fluid dynamics, morphological characteristic, hemodynamic index distribution

## Abstract

The modular inner branched stent-graft (MIBSG), a novel interventional therapy, has demonstrated good effects in the endovascular treatment of aortic arch pathologies, especially those involving the supra-aortic branches. Nevertheless, the long-term efficacy of the MIBSG and in-depth quantitative evaluation of postoperative outcomes remain to be examined. Moreover, the regularity of postoperative vascular remodeling induced by MIBSG implantation has yet to be explored. To address these questions, we constructed four models (normal, preoperative, 1 week postoperative, and 6 months postoperative) based on a single patient case to perform computational fluid dynamics simulations. The morphological and hemodynamic characteristics, including the velocity profile, flow rate distribution, and hemodynamic parameter distribution (wall shear stress and its derivative parameters), were investigated. After MIBSG implantation, the morphology of the supra-aortic branches changed significantly, and the branch point moved forward to the proximal ascending aorta. Moreover, the curvature radius of the aortic arch axis continued to change. These changes in morphology altered the characteristics of the flow field and wall shear stress distribution. As a result, the local forces exerted on the vessel wall by the blood led to vessel remodeling. This study provides insight into the vascular remodeling process after MIBSG implantation, which occurs as a result of the interplay between vascular morphological characteristics and blood flow characteristics.

## 1. Introduction

Aortic arch pathologies (AAPs), such as aortic arch dissection and aortic arch aneurysm, are sometimes associated with severe outcomes. Endovascular aortic repair (EVAR) is widely used as a minimally invasive treatment for AAPs in clinical practice [1,2,3,4]. With advances in instrument technology and the accumulation of clinical experience, endovascular technology for AAPs has made great progress. The branched stent, fenestration stent, and other technologies have been developed [2,5,6,7,8]. However, challenges remain due to the curved structure of the aortic arch and the need to preserve the important brachiocephalic branches of the arch. Branched graft techniques require custom-made devices and complex procedures, which makes them less promising in cases requiring urgent EVAR [9,10,11,12]. For example, the gap between the branch stent and the main stent increases the incidence of postoperative leakage complications [13,14]. Moreover, fenestrated stent technology requires the adjustment of conventional aortic grafts based on the anatomical morphology of the aortic arch. During release, the preopening window of the graft is aligned with the opening of the branch vessels, which increases the procedural difficulty [15,16].

**Figure 1:**
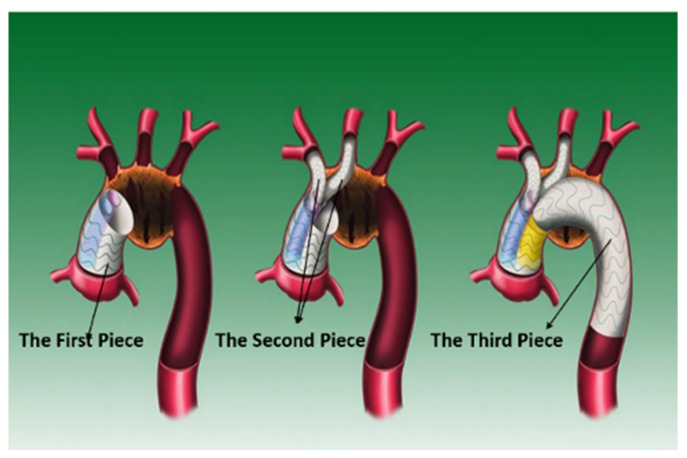
Diagram of the MIBSG.

The modular inner branched stent-graft (MIBSG) is a novel stent with an innovative design and a simple release method [17,18,19] that can be used to reconstruct the aortic arch and its upper branches. The MIBSG system comprises three modules, as shown in Figure 1. The first module is a straight stent-graft with double inner branches anchoring to the ascending aorta. The second module comprises covered stents that can be used to reconstruct the superior arch branch. The third module is a flexible thoracic stent-graft that is intended to isolate the aneurysm. The stent-graft is designed using modularization techniques, which makes it more adaptable (the stent is not patient-specific; it is an off-the-shelf application) with an uncomplicated implantation procedure. The technical feasibility of the MIBSG has been verified in animal experiments and clinical trials. Dozens of patients have been treated with this approach, and the early postoperative results are satisfactory. However, to date, the clinical evaluation of MIBSG efficacy has been based on imaging evaluation, which is insufficient for long-term assessment. Nevertheless, computational fluid dynamics (CFD) provide a promising alternative.

While physiological indicators, such as blood perfusion are the concern of physicians, hemodynamic researchers care about the flow field characteristics related to specific pathologies, such as aortic arch aneurysm and type B aortic dissections, as well as the normal flow field characteristics. 

CFD is extensively used to assess hemodynamic parameters, such as flow velocity, blood pressure, and wall shear stress (WSS), to analyze the long-term efficacy of EVAR. With CFD, blood vessels before and immediately after surgery are reconstructed using clinical image data. Then, the reconstructed models are used for CFD or fluid—structure interaction computations. Using this approach, the efficacy of surgical treatment or other assessments can be carried out based on the analysis of the numerical results. 

In addition, sometimes CFD is the only choice if we need to elucidate the influent of a specific factor on the development of the course of disease. For example, as well known, the functions of stent-graft are to reinforce the wall of an aneurysm to prevent it from rupture, or to seal the tear in the aorta in the case of a dissection. In some cases, the stent-graft does not fit perfectly with the aortic wall, and a so-called bird-beak configuration forms. The bird-beak effect may lead to reduction in the lumen patency, and its long-term influence on the pressure drop and WSS-based parameters is revealed by the usage of CFD-based methods [20,21].

Moreover, CFD research on other important factors, such as the aortic diameter [22] and the beginning position for an octopus endograft [23], are helpful to the clinical predictions.

Similarly, more complicated studies on the surgical intervention techniques are performed to analyze their advantages and disadvantages to a specific patient before surgery [24,25].

While the blood flow field has important physiological meanings, it is attractive to extract an “ideal” structure from real tissues to study the effects of the predominant structure characteristics only [26,27]. This approach is significant for the understanding of typical flow patterns in tissues, such as secondary flow and helical flow.

It is also important to note that vascular remodeling after surgery is a long-term process. The geometric characteristics of blood vessels change continuously over time, which also causes changes in hemodynamic characteristics [28]. Therefore, the characteristics of intraluminal blood flow immediately after interventional therapy are not the sole predictor of long-term outcomes. 

In this study, we evaluated the hemodynamic characteristics of a single case of MIBSG implantation. A preoperative model and two postoperative models (immediately and 6 months after MIBSG implantation) were reconstructed from computed tomography angiography (CTA) images. An optimized normal geometry model was used as a control. Using CFD, flow quantities were extracted, and their distributions were examined by referring to geometric changes to explore the interactions between vascular morphology and hemodynamics.

## 2. Materials and Methods

### 2.1. Image-Based Model Reconstruction

**Figure 2:**
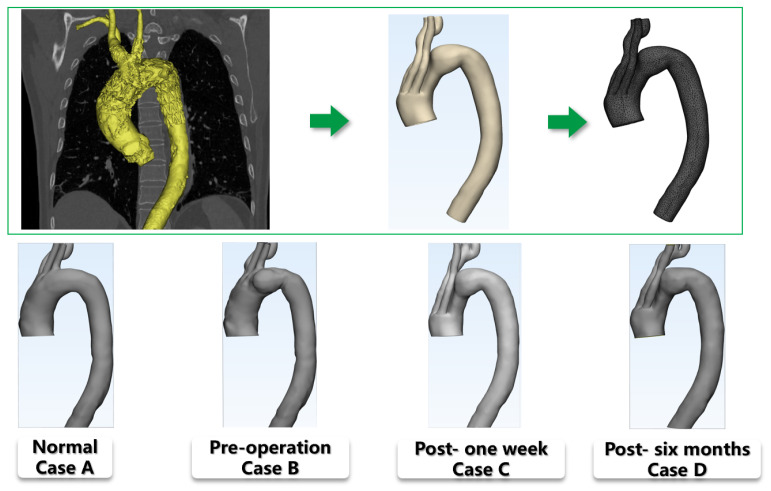
Top: Three-dimensional model reconstruction and mesh generation. (Case **A**) Normal geometry; (Case **B**) Preoperative geometry; (Case **C**) 1-week postoperative geometry; (Case **D**) 6-month postoperative geometry.

The patient, a 74-year-old male, suffered from hypertension and presented with a cystic aortic arch aneurysm. The aneurysm was excluded, and two branches of the innominate artery (IA) and the left common carotid artery (LCCA) were reconstructed by MIBSG implantation. The left subclavian artery (LSA) was occluded after surgery, and LCCA-LSA bypass could take effect when needed (Figure 2). 

Based on CTA images, the three-dimensional geometry was reconstructed using Mimics commercial software (version 19.0; Materialise, Plymouth, MI, USA). The geometry of the MIBSG was not reconstructed separately, but the composite structure of MIBSG-aortic arch was reconstructed as a whole. This is due to the fact that the simulation used in this research is pure CFD. On the other hand, the reconstruction of stent-graft from postoperative CTA datasets is technically difficult. The preoperative and postoperative geometry (1 week and 6 months after MIBSG implantation) were reconstructed with a supra-arch branch wall thickness of 1.0 mm and an aortic wall thickness of 2.0 mm. To illustrate the utility of MIBSG implantation in restoring the normal flow pattern, a normal geometry model was constructed with the aneurysm removed (Figure 2). All of the models started above the sinus of the artery and ended at the bifurcation point of the renal artery.

### 2.2. Computational Framework

The blood flow through the aortic arch was modeled as laminar flow and simulated using the laminar flow module of commercial finite element analysis software (COMSOL 5.5). Generally, turbulent flow patterns are always observed in the blood flow inside the aneurysm and the proximal or distal portion of the aortic arch due to the complex geometry structure and relatively high velocity, i.e., large Reynolds number. Therefore, the use of turbulent models, such as Reynolds-averaged Navier-Stokes (RANS) or large eddy simulation (LES) and even direct numerical simulation (DNS) is natural in hemodynamic researches [29,30]. However, physiologically, the turbulent flow in aorta is harmful, and the high degree of pulsatility of blood flow depresses the development of turbulence [31]. Moreover, research by Xu et al. [23] showed that the laminar flow model with adequately fine mesh resolutions near the walls is capable of capturing the main flow patterns. Therefore, in this research, the laminar flow model is employed with six boundary layers to capture the near-wall blood flow behaviors. Mesh-independent solutions were validated using sufficiently fine elements, for example, 795,698 tetrahedral elements were used for the normal model. The time step used by the implicit solver is adaptive with the maximum value of 0.05 s and the relative error is set to 0.005 between two inner iterations. 

The blood is naturally a non-Newtonian fluid; however, in aortas, its viscosity changes slightly and thus a Newtonian constitutive relationship is used for the blood. The incompressible Navier-Stokes equation was used to describe its flow behavior. The density and viscosity were set at 1060 kg/m^3^ and 0.0035 Pa·s, respectively.

### 2.3. Boundary Conditions 

**Figure 3:**
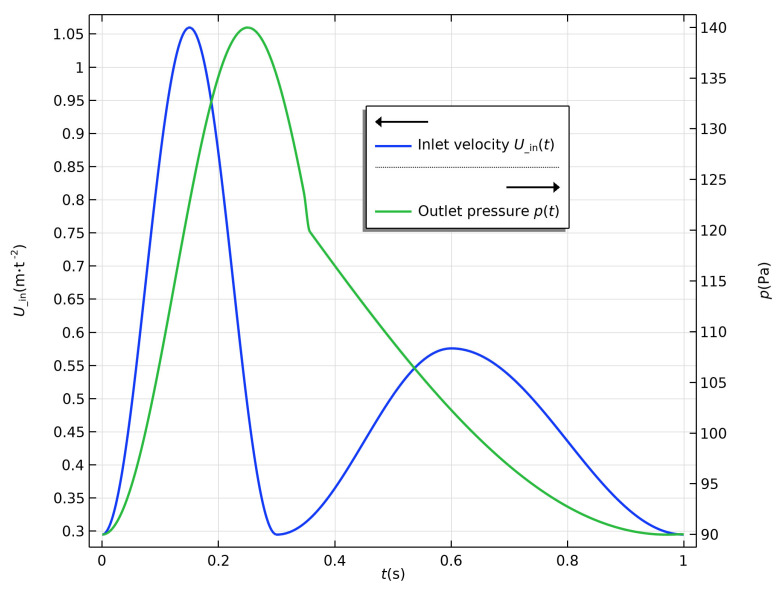
Inlet and outlet boundary conditions.

In the simulation, the outer wall of the aortic arch was fixed, which induced displacement of the order μm of the inner wall, which had a trivial effect on the characteristics of the flow field. Therefore, the inner walls were maintained as rigid in all four simulations, and the two-way fluid—structure interaction between the blood and vessels was dismissed. Blood flow in the aorta is pulsatile, thus the boundary conditions of the inlet and outlet must be set accordingly. Considering the convergence of CFD problems and physiological properties, velocity condition was used as the inlet and pressure condition was used as the outlet in all four models. The inlet and outlet were extruded outward to five-times their diameter to develop the flow pattern. The velocity condition profile was based on the Doppler ultrasound measurement, and the pressure condition was derived from the clinical examinations. The expressions for velocity condition and pressure condition were as follows:

(1) $$v\left(t\right)=\left\{\begin{array}{c} \begin{array}{c} -0.3825\cos\left(6.6667\pi t\right)+0.6775,\;\;\;\;\;\;\;\;\;\;\;\;\;\;\;\;0<t\leq 0.3\;s\\ -0.1405\cos\left(3.3333\pi \left(t-0.3\right)\right)+0.4355,\;\;\;\;0.3<t\leq 0.6\;s\\ -0.1405\cos\left(2.5\pi \left(t-1\right)\right)+0.4355,\;\;\;\;\;\;\;\;\;\;\;\;\;0.6<t\leq 1\;s \end{array} \end{array}\right.$$

(2) $$p\left(t\right)=\left\{\begin{array}{c} \begin{array}{c} -25\cos\left(4\pi t\right)+115,\;\;0<t\leq 0.35\;s\\ \left(p\left(0.35\right)-90\right)\cos\left(0.7692\pi \left(t-0.35\right)+\pi -1.5\right)+p\left(0.35\right),\;\;0.35<t\leq 1\;s \end{array} \end{array}\right.$$

where *v* and *p* are velocity and pressure, respectively. Their profiles are shown in Figure 3.

### 2.4. Related Indicators

Hemodynamic parameters, such as time-averaged wall shear stress (TAWSS), oscillatory shear index (OSI), and relative residence time (RRT), were derived from the WSS. The distributions and trends of these parameters provide clues to the evolution of the restored flow patterns, which then cause the physiological response of cells to the shear stress and strain exerted by blood. Therefore, hemodynamic parameters carry important physiological meanings, and they serve as a bridge between CFD and clinical practice. In this study, hemodynamic parameters were calculated in the post-process to assess the treatment efficacy.

TAWSS is defined as a scalar that indicates the time-averaged magnitude of the surface shear force, as follows:

(3) $$\mathrm{TAWSS}=\tau_{\mathrm{abs}}=\frac{1}{T}\int_{0}^{T}\left|\vec{\tau_{w}}\right|dt$$

Taylor, Hughes, and Zarins [32] defined another scalar as the magnitude of the time-averaged mean shear force, as follows:

(4) $$\tau_{\mathrm{mean}}=\left|\frac{1}{T}\int_{0}^{T}\vec{\tau_{w}}dt\right|$$

Then, the OSI was calculated using the above two scalars, as follows:

(5) $$\mathrm{OSI}=\frac{1}{2}\left(1-\frac{\tau_{\mathrm{mean}}}{\tau_{\mathrm{abs}}}\right)$$

Finally, the RRT was evaluated using the TAWSS and OSI, as follows:

(6) $$\mathrm{RRT}=\frac{1}{\mathrm{TAWSS}\left(1-2\;\mathrm{OSI}\right)}$$

## 3. Results

### 3.1. Morphological Description

The morphology of the branches above the aortic arch changed after MIBSG implantation. As shown in Figure 2, the IA and LCCA branches were reconstructed, and the LSA was sealed after surgery (Figure 2). The ostia of supra-arch branches were located in the first module of the MIBSG, moved from the preoperative arch to the nearby proximal end.

**Figure 4:**
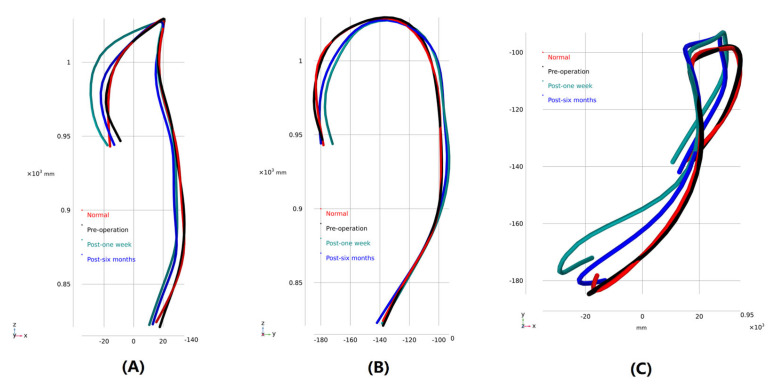
Vessel centerline (contains no branches). (**A**) Coronal plane view; (**B**) 90° anti-clockwise rotation of coronal plane view; (**C**) Horizontal plane view.

To explore the geometric characteristics of the aortic arch at different stages, Mimics software was used to capture the vessel centerlines of the models (the branches were uncontained). The morphological characteristics of the aorta were analyzed at the three observational stages and in the normal model. The highest points of the height direction of the models were set to overlap each other for comparison (Figure 4). The centerline direction of the aortic arch changed greatly owing to the existence of the stent-graft. The radius of the aortic arch curvature significantly decreased 1 week after MIBSG implantation, but it increased after 6 months.

### 3.2. Blood Perfusion

**Figure 5:**
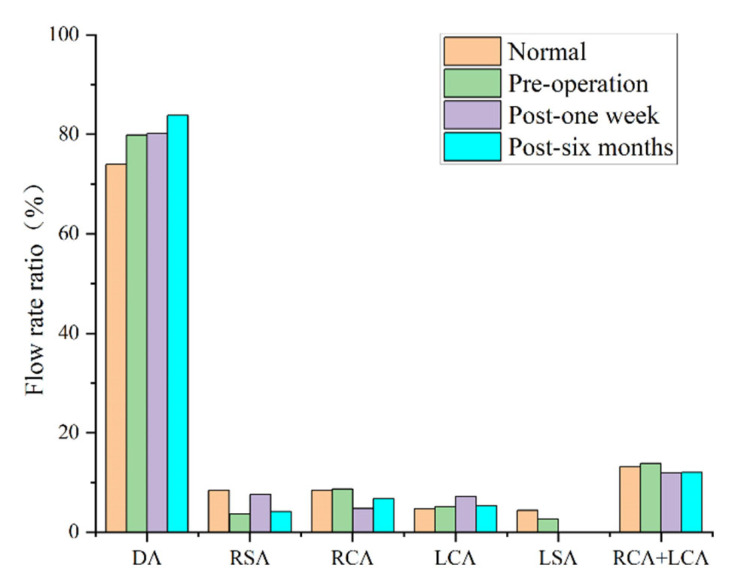
Average perfusion rate of each outlet within one pulsation cycle.

The perfusion rate has always been a consideration for cardiovascular surgery clinicians. In this study, the inlet and outlet flow rates were calculated via surface integration. Figure 5 shows the average perfusion rate of each outlet within one pulsation cycle. Figure 5 shows that, compared with the perfusion rate of the normal model, the existence of an arch aneurysm reduced the total perfusion rate of the superior arch branch. There was also a corresponding decrease in the total volume of the two branches (right common carotid artery [RCCA] + LCCA), which supplies blood to the brain. Six months later, the total perfusion rate of the superior arch branch significantly decreased, especially for the right subclavian artery (RSA) branch. The perfusion rate of the RCCA significantly decreased, and the RSA was compensatory. 

### 3.3. Hemodynamic Description

Flow pattern

**Figure 6:**
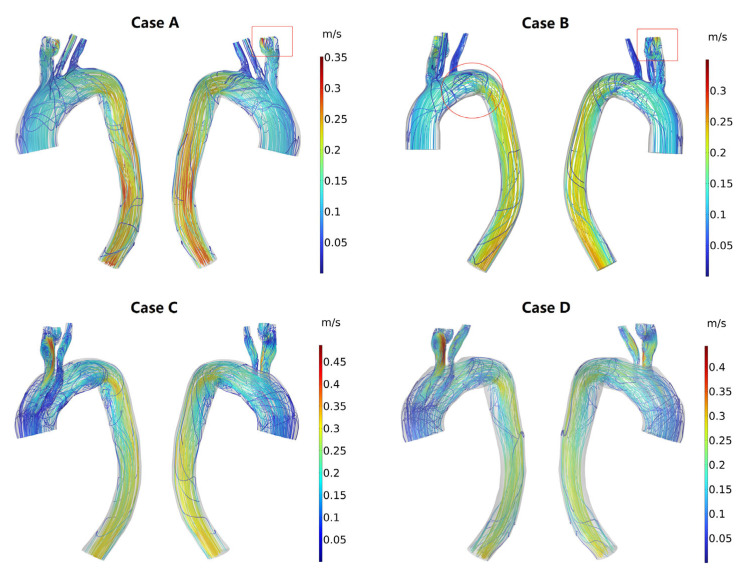
Intravascular flow field at peak systole. (Case **A**) Normal model; (Case **B**) Preoperative model; (Case **C**) 1-week postoperative model; (Case **D**) 6-week postoperative model. The dashed circle is proximal to the third module and adjacent to the overlapping area of the branched stent and aortic stent. The red arrow indicates an area of disturbed blood flow along the lesser curvature of the aorta.

The profiles of peak systolic streamlines visibly highlight the flow patterns of the models at different stages after MIBSG implantation (Figure 6). Compared with Case A (the normal model), in Case B (preoperative), the aneurysm had a larger impact on the blood flow pattern, there was a clear turbulent flow in the aneurysm, and the disturbed flow in the proximal lesser curvature of the descending aorta was strengthened (Figure 6). Additionally, a clear backflow is observed inside the aneurysm. After treatment, the branched stent overlapped with the aortic stent in the first module and was compressed. The influence of this geometric feature on blood flow was as follows. First, the location and lengthening of the branches strengthened the disturbed blood flow within the branches. Second, clear blood flow disturbance could be seen at the proximal end of the third module. Third, there were more vortices of varying intensity in the lesser curvature of the descending aorta at both postoperative stages for the increased curvature, and thus strengthened the secondary flow here (1 week [Case C] and 6 months [Case D]; Figure 6).

Pressure

**Figure 7:**
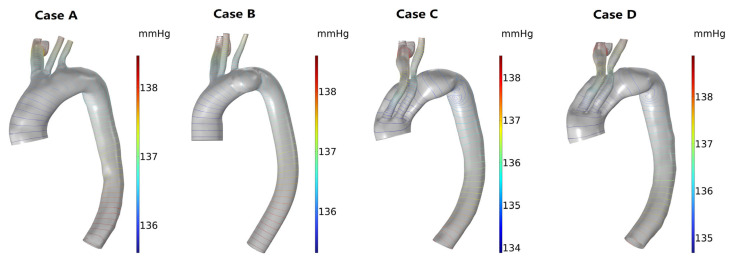
The pressure distribution of the four models at peak systole. (Case **A**) Normal model; (Case **B**) Preoperative model; (Case **C**) 1-week postoperative model; (Case **D**) 6-week postoperative model.

No significant pressure distribution differences were observed between the model with the presence of an arch aneurysm and the normal model; however, a uniform decrease in pressure existed in both models. One week after MIBSG implantation, the decrease in pressure of Case C increased, and an area with an adverse pressure gradient in the lesser curvature of the proximal descending aorta appeared. However, the overall decrease in pressure of the model 6 months after MIBSG implantation was smaller than observed 1 week after MIBSG implantation, and the adverse pressure gradient on the side of the lesser curvature of the proximal descending aorta was also relieved (Figure 7).

WSS-based hemodynamic indicators

WSS-based hemodynamic indicators, such as time-averaged TAWSS, OSI, and RRT, have important physiological meanings. According to Malek [33], regions with a TAWSS of <0.4 Pa, an OSI of >0.25, and an RRT of >5 Pa^−1^ show a stronger tendency to develop arterial atherosclerosis. In an attempt to clearly distinguish the tendency of different postoperative complications, high-risk regions with abnormal values for these factors were classified. 

**Figure 8:**
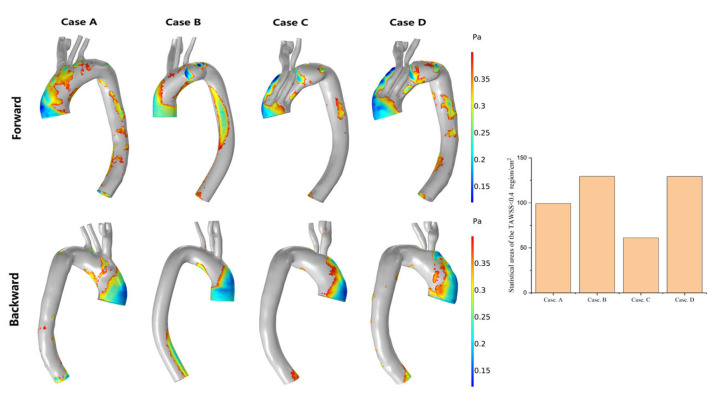
Changes in the regional distribution of the TAWSS of <0.4 Pa and the area statistics of the four models. (Case **A**) Normal model; (Case **B**) Preoperative model; (Case **C**) 1-week postoperative model; (Case **D**) 6-week postoperative model.

Figure 8 shows the distribution of areas with a TAWSS of <0.4 Pa in the four models. Compared with the model with a normal structure, the area with a TAWSS of <0.4 Pa increased in the presence of an aneurysm, and the affected area was located mainly in the aneurysm and lesser curvature of the descending aorta. One week after MIBSG implantation, this area decreased in size and was even smaller than in the normal model. However, 6 months after MIBSG implantation, this area increased in size and was primarily distributed in the third module stent and descending aorta. One week and 6 months after MIBSG implantation, the area with a TAWSS of <0.4 Pa appeared on every branch, which was not apparent in the preoperative and normal models.

**Figure 9:**
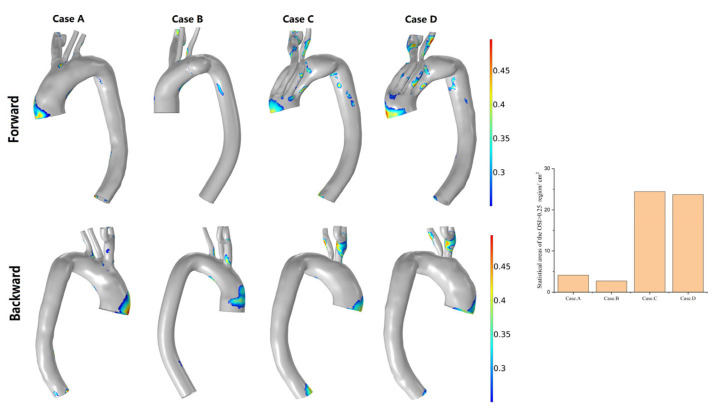
Changes in the regional distribution of the OSI of >0.25 and the area statistics of the four models. (Case **A**) Normal model; (Case **B**) Preoperative model; (Case **C**) 1-week postoperative model; (Case **D**) 6-week postoperative model.

The higher the OSI, the higher the intensity of the shear stress oscillation due to fluctuations of flow. The regional distribution of an OSI of >0.25 and the area statistics in the four models are shown in Figure 9. The area with an OSI of >0.25 was smaller in the normal model, and it was predominantly distributed in regions with disordered blood flow, such as areas proximal to the great curvature of the ascending aorta and vascular branch bifurcation (Case A in Figure 9). The presence of an aneurysm decreased the overall area with an OSI of >0.25 but increased the area with an OSI of >0.25 on the branching vessels. The area with an OSI of >0.25 increased significantly after stent implantation. The distribution characteristics of these regions were analogous in the 1-week and 6-month postoperative models. The region with a high OSI increased clearly in the proximal part of the third module, the proximal part of the lesser curvature of the descending aorta, and the two branch vessels of the two postoperative models (Cases C and D of Figure 9).

**Figure 10:**
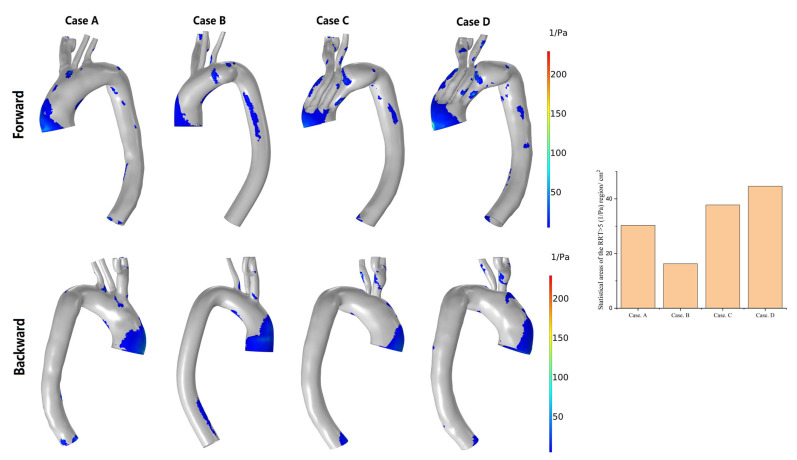
Changes in the regional distribution of the RRT of 5 Pa^−1^ and the area statistics of the four models. (Case **A**) Normal model; (Case **B**) Preoperative model; (Case **C**) 1-week postoperative model; (Case **D**) 6-week postoperative model.

A higher RRT (particle retention time) indicates that the physical material in the blood vessel stays there longer and may eventually form deposits. As shown in Figure 10, the distribution characteristics of regions with an RRT of >5 Pa^−1^ are similar to those with a TAWSS of <0.4 Pa. This suggests a long residence time of substances in the blood in these regions.

## 4. Discussion

As a new stent technology, MIBSG with novel design was proposed to address two main problems: Poor tolerance of brain tissue to ischemia and the reconstruction difficulty caused by a large arch curvature. Despite good clinical imaging results after MIBSG, there is still no detailed analysis of local hemodynamics to predict the outcomes of endovascular surgery. To fill this gap in knowledge, we examined the postoperative blood flow field using image-based CFD. The blood flow characteristics pre- and post-EVAR have been previously analyzed to evaluate the pathological development and postoperative efficacy [21,34,35,36,37,38,39]. Here, we combined morphological characteristics and computational hemodynamics to provide a detailed analysis of the impact of MIBSG implantation in the follow-up period of a single patient. 

For numerical simulation, the geometry profile of the solution domain provides the boundary of a fluid dynamic problem, and thus imposes a remarkable influence on the flow characteristics, e.g., the flow pattern [40,41,42]. This drives the need of an accompanying analysis of the morphology evolution when the analysis of flow patterns is performed.

The aortic arch is complex for its curvature in three dimensions, and the aneurysm enlarges the local diameter of the aortic arch. According to fluid dynamic theory, the bend of the arch generates a secondary flow [43,44]. The secondary flow occurs due to the velocity difference between the lesser and greater curvature of the arch and thus the difference of centrifugal forces when the blood flows through the aortic arch. This indicates that the strength of the secondary flow is related directly with the curvature of the aortic arch. This is why the vortex region was more evident on the lesser curvature side of the descending aorta in the Case C model (Figure 6), compared to the preoperative model. This is one of the factors that gives rise to the risky areas, which consists of great physiological meanings. 

In addition to the bending, the aortic arch has significant torsion, which leads to the helpful helical flow.

Another notable morphology feature is the ostia of supra-arch branches, which also consists of the bifurcation sites of the blood flow. As blood enters the bifurcation sites, the flux distributes among the branches. The ostia of supra-arch branches moved from the preoperative arch to the nearby proximal end after surgery (Figure 2), i.e., the bifurcation sites changed greatly. Another change in the ostia is that the LSA is blocked. These factors cause the blood perfusion to change significantly, as shown in Section 3.2. When the blood enters the branch, flow separation may occur, resulting in low wall shear stress regions [31].

With respect to the aneurysm, for its growth deviation from the main flow direction, an adverse pressure gradient arises near the neck of the aneurysm, which gives birth to a vortex [22,44,45], and results in the slow speed of blood flow. Additionally, the longer resident time of blood particles in this region (Case B) provides another formative factor for the risky areas.

As discussed above, after the treatment of AAPs, the morphologies of the aortic arch change significantly, which alters the existing flows. This provides a clue for the assessment of different treatment approaches before operation [13,20,22,23,26,28]. 

Another aspect to be noticed in the treatment of AAPs with stent-graft is that a difference in the rigidity of the stents and adjacent vessels creates a mismatch between their displacement [46,47,48,49,50]. Moreover, stent-graft placement inside the aortic arch modifies the aortic curvature [21,51], and the mechanical interaction between the vascular wall and the stent-graft may cause changes in anatomical features [52]. The change in vascular morphology and the consequent hemodynamics, which are greatly affected by the anatomy, greatly influence the long-term outcomes of stent placement [21,53]. In this study, we found that the central axis of the aortic arch changed after stent implantation. The radius of the aortic arch curvature was significantly smaller 1 week after MIBSG implantation than in the normal model and the preoperative model. This geometrical characteristic increases the blood flow resistance, which is reflected by the larger pressure decrease in the whole model of Case C (Figure 7). The change in blood flow characteristics; the mismatch in mechanical properties, such as Young’s modulus and Poisson’s ratio; and displacement between the stent and blood vessel can cause continuous blood vessel remodeling. As a result, the radius of the curvature of the central axis of the blood vessel increased 6 months after surgery (Case D) compared with 1 week after surgery (Case C), which indicates that the stress within the vessels and MIBSG is released to some extent. Additionally, clear changes in the blood perfusion rate of each supra-aortic branch were observed as shown in Figure 5.

## 5. Limitations

This study has some limitations that should be noted. First, only a single case of MIBSG implantation was analyzed. Therefore, more cases should be assessed to explore the regularity of vascular remodeling after MIBSG implantation. Second, only the blood flow field is investigated using the CFD technique. The deformation of MIBSG and the aortic arch, the interaction between MIBSG and aortic arch, i.e., the mechanical mismatch between them, the interaction between MIBSG and blood flow, and between the aortic arch and blood flow are left out of consideration. This neglection is to some extent reasonable if our focus is on the main flow characteristics. On the other hand, if we need to assess the long-term durability of MIBSG, its interactions with the aortic arch and the blood flow are necessary to be taken into account. Third, the same boundary conditions were used in all four models, which is ideal and cannot capture the real clinical scenario. This is attributed to the difficulty of measuring the velocity profile and pressure distribution of arbitrary vessel cross-sections in vivo. Without doubt, velocity and pressure conditions at the cross-section of the inlets and outlets are crucial for the numerical results of CFD. Therefore, more non-invasive measurement methodologies and instruments with high resolutions are needed, such as 4D-flow MRI. It is possible to retrieve the velocity of any point within the scan, whereas the resolution near the vessel walls is still not satisfying. Compared with other measurements, the flow field visualized by 4D-flow MRI provides more reliable boundary conditions and can be used to calibrate the results of CFD. We have tried to apply this methodology in this research in the early stage, but image artifacts restricted its reliability. This is to be addressed in further research.

## 6. Conclusions

In this study, CFD was used to assess the long-term efficacy of MIBSG implantation. Compared with the preoperative model, the flow pattern of the postoperative models changed greatly, with more vortices in the lesser curvature of the descending aorta. Compared with the trend in hemodynamic parameters in the model immediately after surgery, the hemodynamic parameters of subsequent models showed an apparent fallback, which indicates that remodeling of the aortic arch morphology was present after surgery.

## Data Availability

The original data were available from the corresponding author upon an appropriate request.

## Abbreviations

CFD	Computational fluid dynamics
AAPs	Aortic arch pathologies
CTA	Computed tomography angiography
IA	Innominate artery
LCCA	Left common carotid artery
RCCA	Right common carotid artery
LSA	Left subclavian artery
RSA	Right subclavian artery
MIBSG	Modular inner branched stent-graft
OSI	Oscillatory shear index
RRT	Relative residence time
TAWSS	Time-averaged wall shear stress
EVAR	Endovascular aortic repair
WSS	Wall shear stress
